# The dangers to children from coconut tree trauma, in KiraKira, Solomon Islands: a retrospective clinical audit

**DOI:** 10.1186/s13690-016-0125-0

**Published:** 2016-04-11

**Authors:** Rajan Rehan, Peter D. Jones, Hashim Abdeen, Heddi Rowas, Jasryn Dhaliwal

**Affiliations:** Faculty of Health Sciences & Medicine, Bond University, Gold Coast, Queensland 4229 Australia

**Keywords:** Falls, Coconut tree, Trauma, Solomon islands, Children

## Abstract

**Background:**

Kirakira is small community of 3,000 people and is the capital of Makira-Ulawa province in Solomon Islands. Kirakira is an impoverished community with a small 30 bed hospital with limited resources. This audit was conducted by final year students from Bond University as part of a selective clinical placement.

**Methods:**

The audit included admissions to the hospital from 2011 to 2014. Trauma-related admissions were identified and classified according to the patient’s age, sex, description of injury, mechanism of injury and whether they were transferred to the National Referral Hospital (NRH) in Honiara for further treatment. Injuries due to Coconut tree trauma were classified as being due to falls from the tree, or trauma from either falling branches or falling coconut fruit.

**Results:**

There were 3455 admissions and 23(0.7 %) non-neonatal deaths over the 3 year period. 126(3.6 %) admissions were referred on to the NRH for further treatment. 277 (8.02 %) admissions were trauma-related with 57(21 %) of these referred on to the NRH. 142 (55 %) of the trauma admissions involved children. Coconut Tree trauma was the commonest cause of a traumatic admission to hospital. There were 49 Coconut Tree trauma admissions including 35 from falls, 12 from falling branches and two from falling coconuts. 80 % of Coconut tree trauma involved Males and the median age of those injured was 13. Primary School age children aged 6–14 years were most at risk for Coconut Tree Trauma. 15(31 %) of the Coconut tree trauma admissions were referred to NRH for further treatment.

**Conclusions:**

Coconut Tree Trauma is common in Kirakira and is an important preventable cause of serious injury that particularly affects primary school aged boys in Kirakira, Solomon Islands. A public education campaign that focuses on this at risk age group warning of the dangers of climbing Coconut trees should be considered.

## Background

Falls from trees remains an important yet preventable cause of trauma resulting in morbidity and mortality in resource poor countries [1]. In the settings where this type of trauma is common, the associated health care systems and the capacity to record reliable data is limited. In the South Pacific, where the Coconut palm is ubiquitous, the literature documenting the outcomes of falls in community settings is absent. Previous studies documenting the injuries caused by falls from Trees in the Pacific have come from retrospective audits of cases of trauma referred to the National Hospitals of Solomon Islands and Fiji [2, 3].

**Table 1:** Table outlining the details of the Coconut Tree Injuries

	Number	Referred to Honiara	% Referred to NRH for further treatment	% Male
All Admissions Kirakira Hospital 2011–2014	3455	128	3.7 %	50 %
All Trauma Admissions Kirakira Hospital 2011–2014	277	57	20.6 %	71 %
Trauma Admissions caused by Coconut Tree Injuries	49	15	30.5 %	71 %
Coconut Tree Injury Aged < 14 years	29	11	37.9 %	66 %
Coconut Tree Injury Aged > 14 years	20	4	20.0 %	75 %
Coconut Tree Trauma Caused by Fall from Tree	35	11	37.9 %	66 %
Coconut Tree Injury caused by being Hit By Branch	12	1	8.3 %	83 %
Coconut Tree Injury caused by being Hit by Coconut Fruit	2	0	0 %	100 %

This study is set in the community of Kirakira [4], the capital of Makira-Ulawa province in Solomon Islands. Kirakira is a small town with an estimated population of 3,000 people. The town has the only hospital which provides care for the Island province of Makira-Ulawa and serves a population of 40,000. The Island is located approximately 230 klms South East of Honiara. Makira Island is 100 km long with a maximum width of 30 km. Makira is covered by dense jungle with a mountainous spine up to 500 m in elevation running along its axis. There is a single unsealed road that runs for approximately 50 klms along just over half of the North coast of the Island. People who do not live along this road have to travel by foot through dense jungle or a treacherous journey by Sea in small boats powered by an outboard motor. 85 % of the population remain subsistence farmers and still live a traditional Solomon Islander lifestyle.

The hospital is 30 bed facility and is staffed by two government employed doctors and approximately 30 nursing staff. It is busy hospital which cares for approximately 1150 acute admissions and 600 deliveries per year with the emergency department seeing over 10,000 presentations per year.

In addition to the hospital the primary health care needs of the province are serviced by 16 nurse clinics that are located in the remote villages across the Island. The typical nurse clinic is a two room timber building where patients can receive care and the second room has 2 two timber beds where woman can confine or patients can be observed overnight. The nurses decide if the patient should be referred to Kirakira hospital for further treatment. The town has an airport that is serviced by four commercial flights per week to Honiara that allows for complex cases to be transferred to the National Referral Hospital (NRH) in Honiara.

In 2012 Kirakira was chosen as a clinical placement site for senior medical students from Bond University to complete a clinical placement. Between 2013 and 2015 there have been 25 groups of Medical students complete a 4 week placement in Kirakira. It was observed that injuries from falls from the Coconut tree were a very common reason for presentation to Kirakira hospital . Each student group reported that during their placement they would see several children in the emergency department because of falls from Coconut trees and that many of these children would be admitted to hospital and several of them were being transferred to the NRH because of the severity of their injuries.

This study aimed to document the types of injuries sustained and the proportion of injuries that would require referral on to Honiara for further treatment and the age range of those most at risk of being injured by falls from Coconut trees. The aim of the study was to develop base line data which could be used to guide and monitor the outcomes of a health promotion campaign directed at reducing the rate and associated adverse health care outcomes caused by Coconut Tree Trauma in Kirakira.

## Methods

We conducted a retrospective audit of the emergency department and hospital records of patients presenting to the hospital between July 2011 and July 2014. The available records were assessed by four independent reviewers. The reviewers were final year medical students completing a 3 week selective clinical placement in Kirakira. The decision to limit the data collection to the previous 3 years was made as these were the records readily available and able to be reviewed in the time available to the reviewers.

Base line data included the total number of admissions, births and deaths recorded for each month and year of the audit. In addition the number of subsequent referrals to the NRH for further management was recorded. Kirakira hospital has an admission logbook where all cases admitted to the hospital via the emergency department are recorded.

A diagnosis of trauma was ascertained by reviewing the hand written notes of the doctor or nurse in the medical file by one of the four medical student reviewers. Trauma related admissions were then identified from the total admissions. These were then classified into various categories including: falls, assault, MVA, burns, blunt trauma etc. These were then further classified into types of injuries including: fractures, dislocations, lacerations, infection, soft tissue injury/pain, amputation or burns.

For each trauma related admission, we recorded the patient’s method of referral, age, sex, a description of and attributed mechanism for the injury and subsequent management. The reviewers further assessed injuries attributed to coconut trees in detail. This comprised of falls from the coconut tree as well as injuries caused by falling branches and falling coconut fruit. A detailed audit of the pattern of trauma that resulted from these injuries was extrapolated from the patient admission charts.

In the Emergency Department all presentations were recorded in a hand written ledger by the nurses who provide the initial assessment for all acute presentations. One line of data was recorded for each patient. This data included the diagnosis and if the patient was admitted to hospital. There data also recorded the name of the patient and their date of birth or approximate age. A new page of the ledger was used for each day. There also a summary page of data recording the total number of presentations and admissions for each month. Each ledger would contain 6 months of data. For this audit six ledgers were reviewed to identify all of the admissions to hospital that were recorded as either due to trauma or due to coconut tree trauma.

The files for each of the admitted patients identified by the review of the emergency department registers were then accessed in the medical records department of the hospital. The admission notes were hand written and some files were incomplete. There were several admissions that were recorded as falls and there was insufficient information in the record to identify the mechanism of the fall. In order to be included in the audit the word coconut tree had to be included in either the Emergency Department register or the patient file. The files identified as being due to coconut tree trauma were checked twice by the team of students prior to recording information extracted from the notes in an excel database.

Ethics Permission to conduct the de-identified audit was approved by the Kirakira hospital’s medical management committee to the medical student’s access to the patient files.

## Results

On average 31 patients are seen in the Emergency Department each day with 33,900 presentations to the Emergency Department of Kirakira Hospital during the 3 years that were audited. The records of 3,455 patients admitted to Kirakira Hospital between July 2011 and June 2014 hospital were assessed by the reviewers. 128 patients (3.7 % of admissions) were transferred to the NRH for further treatment. Trauma accounted for 277 admissions (8.0 % of admissions) in the 3 year period. 57 of these admissions (20.6 % of trauma admissions) were referred to the NRH. Trauma was responsible for 44.5 % of all referrals to the NRH. 71 % (197 of 277) of trauma related admissions were male patients.

Coconut Tree related injuries were the single most common cause of trauma related injury resulting in admission to hospital. There were 49 patients in total over the 3 year period, making up approximately 17.7 % of all trauma patients. 35 patients fell from the Coconut tree, 12 were struck by a branch falling from the Coconut tree and 2 younger children, both under the age of five, were struck on the head by a falling Coconut both requiring admission to hospital.

**Fig. 1:**
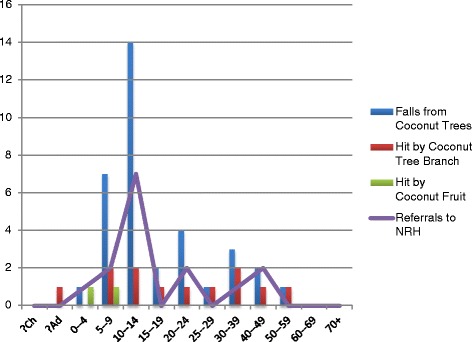
Forty-nine Coconut-Tree Related Episodes of Trauma between July 2011–June 2014. Mechanisms of Injury and proportion referred to the National Referral Hospital according to Age Groups

These details outlining the ages of the admitted patients, the mechanism for their injury and whether the patients were referred to the NRH are shown in Fig. 1 . Males were far more likely to be admitted for Coconut related trauma was 82 % (40 of 49) of presentations being male. 30.5 % (15 of 49) of Coconut tree related trauma admissions was serious enough to require referral on to the NRH. The median age of patients admitted for Coconut Tree related trauma was 13.0 years. 59 % (29 of 49) were children 14 years of age and younger and 38 % (11 of 29) of these children suffered trauma serious enough to warrant referral on to the NRH. The summary data for the audit are presented in Table 1.

**Fig. 2:**
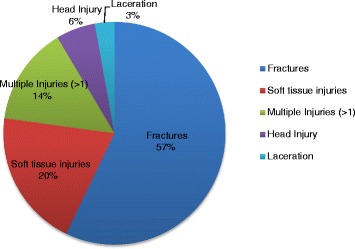
Types Injuries Sustained from 35 patients who Fell from a Coconut Tree July 2011 - June 2014

Figure 2 shows the different types of injuries sustained by those that fell from Coconut Trees. Falls made up 71 % of coconut tree trauma (35 of 49 cases). 57 % (20 of 35) of patients who had suffered a falls from a Coconut tree were diagnosed as having a fracture. Falls and the associated fractures were more likely to require referral on for specialist treatment. This graph documents that these falls can also causes soft tissue injuries, closed head injuries, multiple injuries and lacerations in addition to the well documented problem of fractures.

Being struck by a branch was most likely to cause soft tissue injury and lacerations requiring hospitalization. Only 1 of 12 injuries caused by a falling branch was serious enough to require referral onto Honiara hospital. This appears to have been due to multiple injuries in this patient. Falling branches caused less severe injuries than falling from the coconut tree.

There were only two recorded instances of falling coconut tree fruit causing an injury that led to admission to hospital during the 3 years of 2011 to 2014. The two cases were both small children, who suffered a relatively minor concussion that resulted in admission to Kirakira hospital for observation.

## Discussion

The observation that falls from trees are a common cause of serious trauma in the Pacific has previously been documented in the literature. The previous studies have been based on reviewing the injury databases from the National hospitals of Papua New Guinea, Fiji and Solomon Islands. As has been the case in previous studies this study confirms that young, primary school aged boys are the most likely candidates for falling from the Coconut Tree1 [1–3].

A weakness of this study was that the proportion of clinical notes that were unusable has not been recorded. A further limitation was that those more mild injuries that did not require admission to hospital have not been reported. There are no detailed records kept by the hospital about those patients that were not admitted and able to be managed as outpatients. In Solomon Islands patients have a small exercise book that they retain as their own personal medical record. This is where the doctor is able to record details of any clinical encounters and that information is not retained by the Health Service. This means that there is no current means for retrospectively obtaining detailed data about those coconut tree injuries that did not result in admission to hospital. A future prospective study could be designed to record this information.

This study adds to the literature because it records data from a remote provincial hospital and because the study involved review of the actual case notes was able to distinguish the different mechanisms by which injuries might occur from Coconut trees. These mechanisms include falling branches, falling fruit and falls of patients attempting to retrieve Coconuts at significant heights. Coconut trees grow to an average height of 9 m. It is little wonder that anyone who falls from such a height is at risk of very serious injury. This is the equivalent of to falling from a fourth floor residential balcony.

A recent study of Tree related injuries in Solomon Islands speculated that those referred on to the NRH represented only a small number of cases [5]. In that study there were 1107 admissions nationally recorded in an injury database over an 18 year period due to falls from trees. This is approximately 60 admissions per year to the NRH for falls from trees. The experience in Kirakira suggests that there at least three times as many children admitted to the smaller rural hospitals of Solomon Islands with injuries sustained through falls from coconut trees compared to the number referred on to the National Referral Hospital in Honiara. This would mean that a crude estimate is that there are approximately 200 cases, the majority of whom are children, admitted to hospital each year due to injuries from falls from Trees in Solomon Islands.

Kirakira is the third biggest rural hospital in Solomon Islands [6]. The health workforce and capacity to deliver health care is limited in Kirakira. In Australia, we are accustomed to a multidisciplinary rehabilitation service for clients that are disabled, chronically ill or recovering from a traumatic injury. There is an almost complete lack of allied health services available in Kirakira with only one physiotherapist (employed by the Japanese Government), and very limited equipment. Appropriate sized crutches for children suffering lower limb fractures were not available during the time the audit was conducted. This means that minor trauma-related injury can lead to a steep decline in functional capacity of the patient. In this context, the chances of having a poor outcome from a serious fracture or dislocation, is quite high in Solomon Islands. The importance of remaining physically healthy is particularly vital where the majority of the men in Kirakira will engage in laboring and subsistence farming and being disabled will mean they are not able to adequately provide for their respective families [7].

This makes the prevention of tree related injuries a public health imperative for the people of Kirakira. Delivering appropriate public health messages and designing a campaign that might lead to a reduction in the “Tree Toll” a significant challenge in Kirakira. There is no TV, radio or local print media in Kirakira. There are a myriad of diverse “wantoks” each with their own languages and this makes developing the materials to communicate with the target population difficult [8]. The public health campaigns in Solomon Islands to date have been focused on communicable diseases such as Malaria and TB and vaccine preventable diseases. There has been success in these areas evident in recent government statistics [6] which means that the prevention of falls from Coconut tees could be an appropriate target for an injury prevention campaign.

Establishing a national database that includes both the NRH and the Rural Hospitals that accurately records both the injuries sustained and the outcomes of those injuries is the first step in attempting to reduce the impact of Coconut Tree related trauma in children. Such a campaign might look at improvements in the documentation in the medical records that would assist in the ability to collect accurate clinical data from these remote settings.

A second step is developing culturally appropriate materials to deliver the message about how Coconut Tree trauma might be prevented.

Like all retrospective audits there are significant limitations associated with this study. To begin with, there was no way to establish if all admission records were accounted for, as there are no electronic records and no way to check the final numbers of files assessed. Additionally, not all records were complete, and in some cases lack crucial information regarding the type of injury sustained or the method of injury was missing. There was also a large variation in the amount of detail recorded in regards to the management received by patients. For example, if an x-ray was done, it may have not been recorded. Of note; only admission notes were reviewed, as there were no outpatient notes held at the hospital. In Kirakira, patients keep their own medical record which they then bring to consultations. It is therefore likely that there are a number of less-severe injuries that have been unaccounted for in this audit amongst those presenting to the Emergency Department. The notes for these clinical interactions are recorded in the patient’s own medical record notebook and simply the name and age of the patient and whether they have been admitted is recorded in the Hospital’s Emergency department record.

## Conclusions

Despite these limitations, this study has documented useful clinical information about the number of serious injuries that are occurring to children from data from a remote rural location in Solomon Islands due to falls from Coconut Trees. The data was has obtained by a group of final year students during a 3 week final year clinical placement. In the process of collecting this date there have been areas identified where improvements could be achieved that might assist in achieving improved outcomes achieved patients in Kirakira. The outcomes achieved by this project demonstrate the potential educational and community value of a carefully structured and organized clinical placement in a resource poor setting in the South Pacific [9].

## References

[1] Barrs P, Dakutala P, Doolan M (1984). Falls from tree associated injuries in rural Melanesia. BMJ.

[2] Mulford JS, Oberli I, Tovosia S (2001). Coconut palm related injuries in the Pacific Islands. ANJ J Surg.

[3] Gupta A, Reeves B (2009). Fijian seasonal scourge of mango tree falls. ANZ J Surg.

[4] Kirakira: Solomon Islands. Available at https://en.m.wikipedia.org/wiki/Kirakira Last accessed August 5, 2015.

[5] Negin J, Vizintin P, Houasia P, Martinuik ALC (2014). Barking up the wrong tree: injuries due to falls from trees in Solomon Islands. MJA.

[6] Health Services in Solomon Islands. WHO report 2012 for the Ministry of Health. www.wpro.who.int/health_services/service_delivery_profile_solomon_islands.pdf last accessed on August 5, 2015.

[7] Tahisi S, Cooper C. Wantok Stori, Collaboration and Exchange: Towards the Development of Creative Industries in Solomon Islands Authors. In the Asia Pacific Journal of Arts and Cultural Management, 2012: Vol 9(1)

[8] Gartrell A, Mandersonl, Jennaway M, Fangalasuu J, Dolaiano S, Socio-cultural attitudes to disability in the Solomon Islands: identifying culturally appropriate solutions to disadvantage (2010–2011). From the summary of Research Finding presented at the Disability Research Symposium, Aug 28–29, 2014, Canberra, Australia: The Australian Disability and Development Consortium.

[9] Smith J, Jones P, Fink J. Peer Mentoring; Evaluation of a new model of Clinical Placement in the Solomon Islands undertaken by an Australian Medical School. Rural and Remote Health15:3410 (On-line) 2015 available at www.rrh.org.au.

